# A method for automatic forensic facial reconstruction based on dense statistics of soft tissue thickness

**DOI:** 10.1371/journal.pone.0210257

**Published:** 2019-01-23

**Authors:** Thomas Gietzen, Robert Brylka, Jascha Achenbach, Katja zum Hebel, Elmar Schömer, Mario Botsch, Ulrich Schwanecke, Ralf Schulze

**Affiliations:** 1 Computer Vision and Mixed Reality Group, RheinMain University of Applied Sciences Wiesbaden Rüsselsheim, Wiesbaden, Germany; 2 Computer Graphics Group, Bielefeld University, Bielefeld, Germany; 3 Department of Prosthetic Dentistry, University Medical Center of the Johannes Gutenberg University Mainz, Mainz, Germany; 4 Institute of Computer Science, Johannes Gutenberg University Mainz, Mainz, Germany; 5 Section of Oral Radiology, University Medical Center of the Johannes Gutenberg University Mainz, Mainz, Germany; Universidade de Sao Paulo, BRAZIL

## Abstract

In this paper, we present a method for automated estimation of a human face given a skull remain. Our proposed method is based on three statistical models. A volumetric (tetrahedral) skull model encoding the variations of different skulls, a surface head model encoding the head variations, and a dense statistic of facial soft tissue thickness (FSTT). All data are automatically derived from computed tomography (CT) head scans and optical face scans. In order to obtain a proper dense FSTT statistic, we register a skull model to each skull extracted from a CT scan and determine the FSTT value for each vertex of the skull model towards the associated extracted skin surface. The FSTT values at predefined landmarks from our statistic are well in agreement with data from the literature. To recover a face from a skull remain, we first fit our skull model to the given skull. Next, we generate spheres with radius of the respective FSTT value obtained from our statistic at each vertex of the registered skull. Finally, we fit a head model to the union of all spheres. The proposed automated method enables a probabilistic face-estimation that facilitates forensic recovery even from incomplete skull remains. The FSTT statistic allows the generation of plausible head variants, which can be adjusted intuitively using principal component analysis. We validate our face recovery process using an anonymized head CT scan. The estimation generated from the given skull visually compares well with the skin surface extracted from the CT scan itself.

## Introduction

Facial reconstruction is mainly used in two principal branches of science: forensic science and archaeology. Remains of a human skull act as input to reconstruct the most likely corresponding facial appearance of the dead person to enable recognition. Traditional methods rely on manual sculpturing a moldable substance onto the replica of the unknown skull using anatomic clues and reference data. Claes et al. [1] consider this a highly subjective procedure requiring a great deal of anatomical and artistic modeling expertise. The result is often limited to a single reconstruction, because it is very time consuming. Computer-based methods can provide consistent and objective results and also allow multiple reconstructions using different meta-information, such as age, or weight, because a reconstruction can be accomplished in a short time [1]. In her comprehensive review, Wilkinson [2] reports that there is a lot of criticism on facial reconstruction techniques from scientists, but following the same method both techniques, manual or computer-based, have a rather small degree of artistic interpretation. Wilkinson concludes that achieving anatomical accuracy should be reproducible and reliable, however some stages in the reconstruction process involve a little degree of artistic interpretation.

Computer-aided facial reconstruction methods have been previously proposed in other publications [3–7]. Related work uses different techniques for the underlying registration as well as for the subsequent facial reconstruction. Although not standardized, FSTT measurements play an important role both in facial approximation and craniofacial superimposition methods due to the quantitative information provided [8]. A wide variety of different techniques such as needle probing, caliper or radiographic measurements, or ultrasonographic assessments are used to determine the FSTT, which lead to different results in the FSTT statistics. In addition, 3D imaging techniques such as CT or Magnetic Resonance Imaging (MRI) are employed for this purpose. Driven by the generally lower radiation dose when compared to medical CT, lately Cone Beam Computed Tomography (CBCT) has also been used [9]. In general it is difficult to compare FSTT studies based on CT and CBCT scans. CT scans are taken in supine position whereby CBCT scans can be taken in various positions (sitting, lying down, standing up), which has different gravity effects on the FSTT. CBCT also has the inherent drawback that some landmarks cannot be found in the data sets because it is normally limited to the craniofacial region. Although not backed by numerical data, it is generally advocated to prefer measurements on living individuals over cadavers [8]. In [8], Stephan and Simpson conclude that regardless of the applied technique the measurement error for FSTT assessment is rather high (relative error of around 10%) and that no method so far can be considered superior to any other. In addition, the authors stated that small sample sizes for most of the studies also compromise the degree to which the results from such studies can be generalized.

Generally spoken, measurements based on a few distinct landmark points yield the inherent drawback of providing only a few discrete thickness values. Areas between these distinct measurement points need to be interpolated. A *dense* soft tissue map would yield important information for facial reconstruction. A statistical head model could be fitted to such a dense soft tissue profile thereby providing an estimate of the visual appearance of the person to be identified, based on *statistics* of the sample data.

Turner et al. [3] introduced a method for automated skull registration, and craniofacial reconstruction based on extracted surfaces from CT data that was applied to a large CT data base consisting of 280 individuals in [4]. For registration of a known skull to a questioned one, the authors use a heuristic to find crest lines in combination with a two-step ICP registration followed by a thin-plate spline warping process. The same warping function is applied to the extracted skin of the known skull. Following, from a collection of 50 to 150 warped skin surfaces they use principal component analysis (PCA) to construct a “face-space” with a mean face for the questioned skull. Using the linear combination of the eigenvectors with some a-priori knowledge, such as age and sex, they are able to generate a subset of most likely appropriate appearances for the questioned subject. To this end, both the questioned and the known skull are represented as polygonal meshes and are reduced to their single, outer surface. Thereby, disregarding the volumetric nature of the bony structure in some cases leads to poor fitting results.

The utilization of a deformable template mesh for forensic facial reconstruction was presented by Romeiro et al. [5]. Their computerized method depends on manually identifying 57 landmarks placed on the skull. Based on these preselected landmarks and a corresponding FSTT (obtained from other studies) an implicit surface is generated using Hermite radial basis functions (HRBF). To improve the quality of the result, they use several anatomical rules such as the location of the anatomical planes and anatomical regressions related to the shape of the ears, nose, or mouth. Hence, the quality of their results strongly depends on an appropriate template that properly takes age, sex, and ethnicity into account.

An approach for craniofacial reconstruction based on dense FSTT statistics, utilizing CT data, was presented by Shui et al. [6]. Their method depends on 78 manually selected landmarks placed on the skull, which guide the coarse registration of a template skull to each individual skull, followed by a fine registration using ICP and thin plate splines (TPS). The FSTT measurement is performed for each vertex of the deformed skull in the direction defined by the geometric coordinate. A coarse reconstruction of a face from an unidentified skull is achieved by translating each skull vertex in the defined direction by the length of the FSTT measured at this position. To achieve a smooth appearance six additional points have to be marked manually for guiding a TPS deformation of a template face to the coarse reconstruction. Finally, the recovery of mouth, eyes, and nose has to be performed by a forensic expert, which makes the method not fully automatic.

Shui et al. [7] proposed a method for determining the craniofacial relationship and sexual dimorphism of facial shapes derived from CT scans. Their approach employs the registration method presented in [6], to register a reference skull and face to a target skull respective face. Applying a PCA to the sets of registered skull and skin templates, they derive a parametric skull and skin model. Through analyzing the skull- and skin-based principal component scores, they establish the craniofacial relationship between the scores and therefore reconstruct the face of an unidentified subject. Although the visual comparison of the estimated face with the real shows good results, these results appear to be due to over-fitting. Moreover, the geometric deviation, especially in the frontal part of the face, are mostly around 2.5–5 mm, which indicates rather inaccurate reconstruction results.

Our approach to forensic facial reconstruction is divided into two parts: model generation and forensic facial reconstruction. Unlike most previous methods [3–7] our approach is fully automated, from the initial skull registration up to the final face reconstruction, and thus does not require any manual interaction. Only the initial model generation (preprocessing or training phase) requires a few manual steps. The next section describes the generation of the three models required for our automated facial reconstruction approach: The parametric skull model, the statistic of FSTT, and the parametric head model. In the following sections the automated facial reconstruction process is presented, including the modeling of variants of plausible FSTT distributions for a given skull.

## Model generation

**Fig 1:**
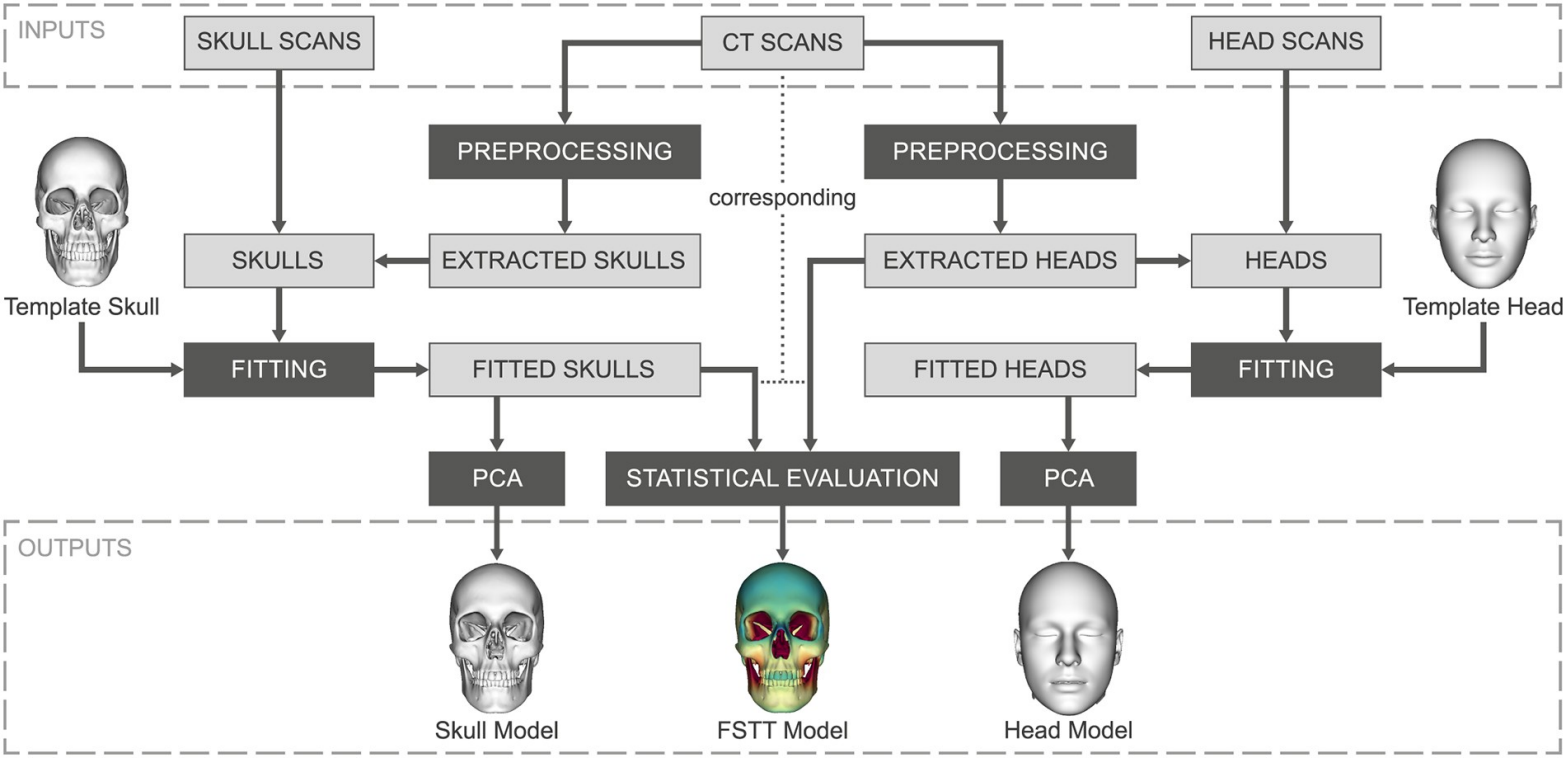
Overview of our model generation processes. Generation of a skull and a head model as well as a dense FSTT statistic from multimodal input data.

In this section we present the proposed model generation processes, as outlined in Fig 1. We use volumetric CT scans and optical 3D surface scans as input and distinguish between two input types: skulls and heads. In the following, the outer skin surface of a head is referred to as *head* and the bony skull structure is referred to as *skull*. In order to obtain a uniform data basis, a *preprocessing* step is performed to extract the skull and the head as triangular surface meshes from each CT scan. In the next step we need to establish the relationship between different skulls as well as between different heads. For this purpose, in a *fitting process*, we register an appropriate template model to each given mesh of a specific input type. After that, we are able to utilize the fitted templates to determine the geometric variability of the skulls respectively heads performing a *PCA*. As result we derive two parametric models: a parametric skull model and a parametric head model. Based on corresponding skulls and heads extracted from CT scans we additionally build a dense FSTT map in the *statistical evaluation* step.

### Database

Following internal ethical review board approval (Ethik-Kommission der Landesärztekammer Rheinland-Pfalz, Deutschhausplatz 2, 55116 Mainz), head CT scans were collected from the PACS system of the University Medical Center Mainz. We only used existing CT data (from four different CT devices) from our database. No subject was exposed to ionizing radiation for this research. The local ethical approval board has approved the processing of the pseudonymized existing CTs (from the DICOM database of the University Medical Center Mainz) to generate the statistical models under the approval number No 837.244.15 (10012) (date: 05.08.2015). In our study we included CT scans that meet the following criteria:

1. The facial skull of the patient is *completely imaged*.
2. The *slice thickness* is less than or equal to 1 mm.
3. The subject has no significant oral and maxillofacial deformations or missing parts.

From several hundred CT scans that we analyzed a total number of 60 were suitable for our purpose. However, only 43 of these scans could be used for generating the parametric head model and the statistic of FSTT, since in the remaining 17 CT scans external forces (e.g. frontal extending neck stabilizers, nasogastric tubes, etc.) compressed the soft tissue. In a *preprocessing* step every CT scan was cropped, such that we obtain a consistent volume of interest limited to the head area. For this purpose the most posterior point of the mandibular bone was determined automatically in the 2D slice images and the volume was trimmed with an offset below this detected position. After this cropping step, bone and skin surface meshes were extracted using the Marching Cubes algorithm [10] (we used the Hounsfield units -200 and 600 as iso-values for skin and bone surface extraction, respectively). To remove unwanted parts, such as the spine or internal bone structures, a connectivity filter was applied to the bone mesh, leaving only the skull. Finally, all extracted meshes were decimated to obtain a uniform point density for all data sets [11]. The meshes extracted from CT data were supplemented by triangle meshes from 3D surface head scans (From www.3dscanstore.com) of real subjects in order to fill up the database for our model generation processes. The 3D surface scans are of high quality, do not suffer from artifacts or strong noise, and consist of about 500 k vertices in case of the head and about 400 k vertices in case of the skull. In summary the following data sets were included in the study:

1. A total number of *p* = 62 skulls (60 extracted skulls from CT scans and 2 skulls from 3D surface scans) were used to generate a skull model.
2. A total number of *q* = 82 heads (43 extracted skin surfaces from CT scans and 39 heads from 3D surface scans) were used to generate a head model.
3. A total number of *r* = 43 corresponding skulls and skin surfaces extracted from CT scans were used to build the FSTT statistic.

### Generating a parametric skull model

In order to generate a parametric skull model we need to establish the relationship between the different skulls from our database. For this purpose, we register a single template skull to each skull individually. This template model has to be a volumetric tetrahedral mesh in order to accurately represent the solid nature of a bony skull. We therefore converted a surface triangle mesh of a skull (Based on www.turbosquid.com/3d-models/3d-human-skull/691781) to a volumetric tetrahedral mesh. Our template skull model, shown in Fig 1, consists of *m* ≈ 70 *k* vertices, whose positions we denote by $S=\{s_{1},\ldots ,s_{m}\}$. Tetrahedra T(S) are built by connecting four vertices each, and the set of all tetrahedra is denoted as T=T(S). The vertices S and tetrahedra T constitute the tetrahedral mesh of our template skull.

The *fitting process* comprises the following two main stages for an input skull with vertex positions $P=\{p_{1},\ldots ,p_{M}\}$:

1. A global rigid transformation that coarsely aligns the input skull to the template skull. The registration starts with the fast global registration approach presented in [12], followed by a refinement step using the well known Iterative Closest Point (ICP) algorithm [13].
2. A fine registration of the template skull to the input skull, which consists of several non-rigid transformation steps, computed by minimizing the energy (inspired by [14])
  $$E\left(S\right)=E_{\text{fit}}(S)+\lambda_{\text{reg}}E_{\text{reg}}\left(S_{\text{prev}},S\right)$$ (1)
  consisting of a fitting term *E*_fit_ and a regularization term *E*_reg_.

In the non-rigid step, the *fitting term*

$$E_{\text{fit}}(S)=\frac{1}{\sum_{c\in C}w_{c}}\sum_{c\in C}w_{c}∥s_{c}-f_{c}{∥}^{2}\;$$

penalizes the squared distance between a vertex on the template skull **s**_*c*_ and its corresponding point **f**_*c*_, which is a point on or close to the mesh of the input skull. The factor *w*_*c*_ ∈ [0, 1] is a per-correspondence weight, which controls the influence of the various correspondences, such as points on the inner or outer skull surface.

The *regularization term*

$$E_{\text{reg}}\left(S_{\text{prev}},S\right)=\sum_{T\in T}(\text{vol}\left(T\left(S\right)\right)-\text{vol}\left(T\left(S_{\text{prev}}\right)\right){)}^{2}$$

penalizes geometric distortion of the template skull during the fitting. $S_{\text{prev}}$ represents the vertex positions of the previous deformation state, while S stands for the current (to-be-optimized) positions. The function vol(*T*) denotes the volume of tetrahedron *T*. Thus, the regularization term penalizes the change of volume of tetrahedra. The non-rigid deformation starts with rather stiff material settings and successively softens the material during the registration process (by reducing λ_reg_).

During the various non-rigid transformation steps we use different strategies to define the correspondences C. First, correspondences are determined by the *hierarchical ICP* approach described in [15], where we register hierarchically subdivided parts of the template skull to the input skull using individual similarity transformations. This results in several small pieces (e.g., the eye orbit) that are well aligned to the input skull. Based on the correspondences found in this step the whole template skull is registered towards the input skull. In subsequent deformation steps, we estimate the correspondences in a closest vertex-to-vertex manner, where we only consider vertices lying in high curvature regions, additionally pruning unreliable correspondences based on distance and normal deviation [15]. In the final non-rigid transformation steps, when the meshes are already in good alignment, we use vertex-to-surface-point correspondences. These correspondences are determined considering all vertices employing a two-step search: First, we search for vertex-to-vertex correspondences from the input skull to the template skull, pruning unreliable correspondences based on distance and normal deviation. Second, we search for correspondences from the computed corresponding vertices on the template towards the input skull. This second step is computed in vertex-to-surface-point manner, this time pruning only large deviation between the vertex and surface normal.

The described two-way correspondence search prevents tangential distortions of the fitted template skull and can handle artifacts in the input skulls, e.g., artifacts in the teeth region due to metallic restorations. Additionally, it makes our registration process robust against the porous bony structure caused by low resolution of the CT scan or the age of the subject. To further prevent mesh distortions we additionally use a release step, where the undeformed template is deformed towards the current deformed state using only preselected points of interest (for further details see [15]).

In order to analyze the accuracy of our skull registration process, we evaluated the fitting error by computing the distance for all vertices of the facial area (which covers all predefined landmarks) of an input skull towards the fitted template model. The mean fitting error for all 62 fitted skulls is below 0.5 mm.

**Fig 2:**
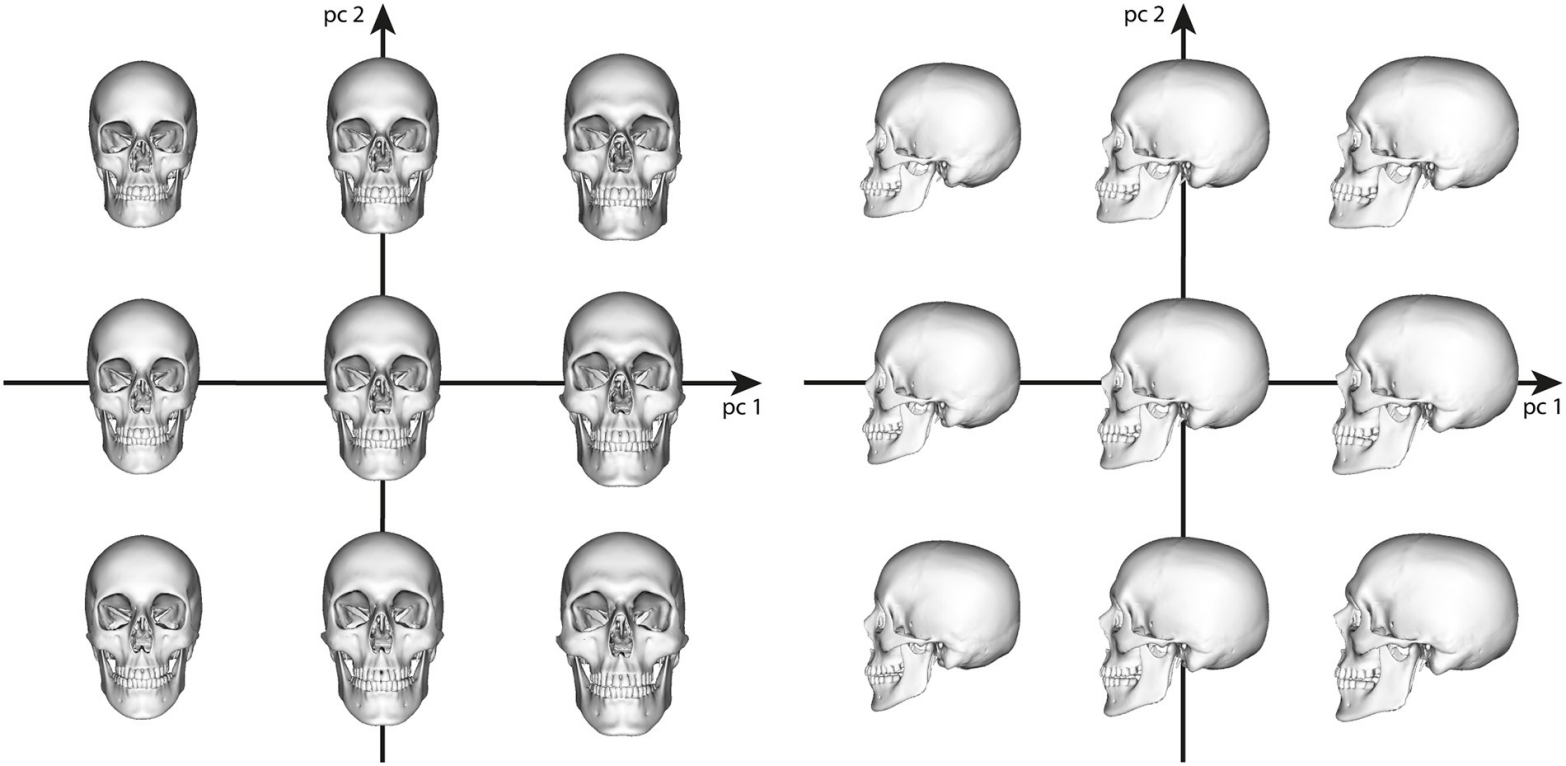
Skull variants along the two principal components with the largest eigenvalues. We visualize $\bar{s}+\alpha_{1}u_{1}+\alpha_{2}u_{2}$, where *α*_*i*_ = *a*_*i*_ ⋅ *σ*_*i*_, *i* = 1, 2, is the weight containing the standard deviation *σ*_*i*_ to the corresponding eigenvector **u**_*i*_, and the factor *a*_*i*_ ∈ {−2, 0, 2}.

Stacking the vertex coordinates of each fitted skull into column vectors **s** = (*x*_1_, *y*_1_, *z*_1_, …, *x*_*m*_, *y*_*m*_, *z*_*m*_)^⊤^ we can apply PCA to the set of fitted skulls (after mean-centering them by subtracting their mean $\bar{s}$). This results in a matrix **U** = [**u**_1_, …, **u**_*p*−1_] containing the principal components **u**_*i*_ in its columns. A particular skull *S* in the PCA space spanned by **U** can be represented as

$$S\left(a\right)=\bar{s}+Ua,$$ (2)

where **a** = (*α*_1_, …, *α*_*p*−1_)^⊤^ contains the individual weights of the principal components of **U**. The parametric skull model (2) can be used to generate plausible skull variants as a linear combination of the principal components, which is depicted exemplarily for the first two main principal components in Fig 2.

We finally select 10 landmarks on the parametric skull model that are used to guide the head fitting process in the automatic forensic facial reconstruction (see detailed explanation in the section on head fitting).

### Generating a statistic of facial soft tissue thickness

**Fig 3:**
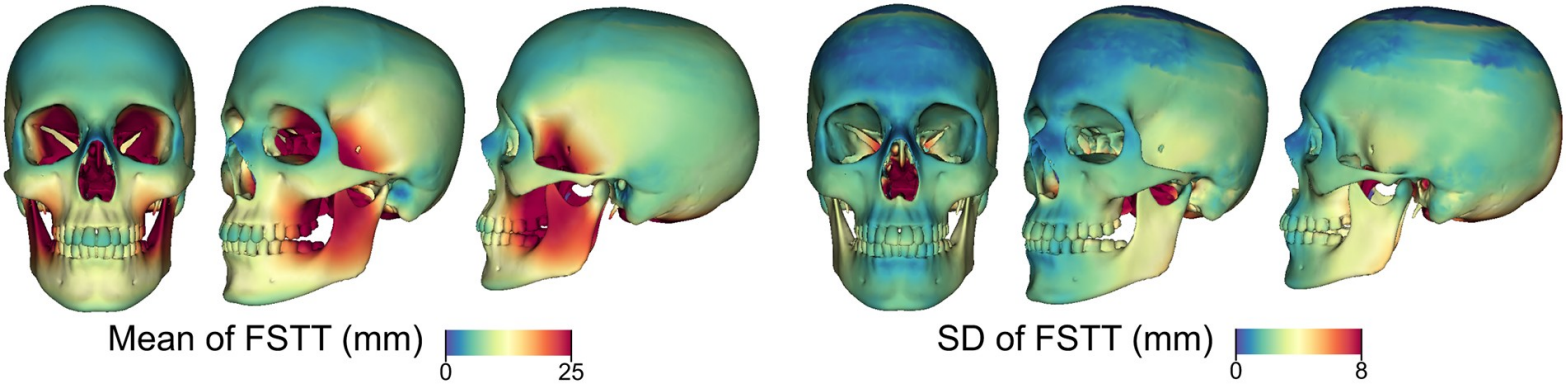
Statistic of the FSTT on a mean skull. Mean and standard deviation of FSTT computed from the 43 CT scans.

In a *statistical evaluation process* the distances between 43 corresponding skulls and heads extracted from the CT scans are measured. To this end, we determine for each vertex of a fitted skull the shortest distance to the surface of the extracted skin surface [16]. Finally, the mean and standard deviation of the FSTT are computed per vertex. Fig 3 shows the mean skull $\bar{s}$ with color-coded mean and standard deviation of the obtained FSTT.

**Fig 4:**
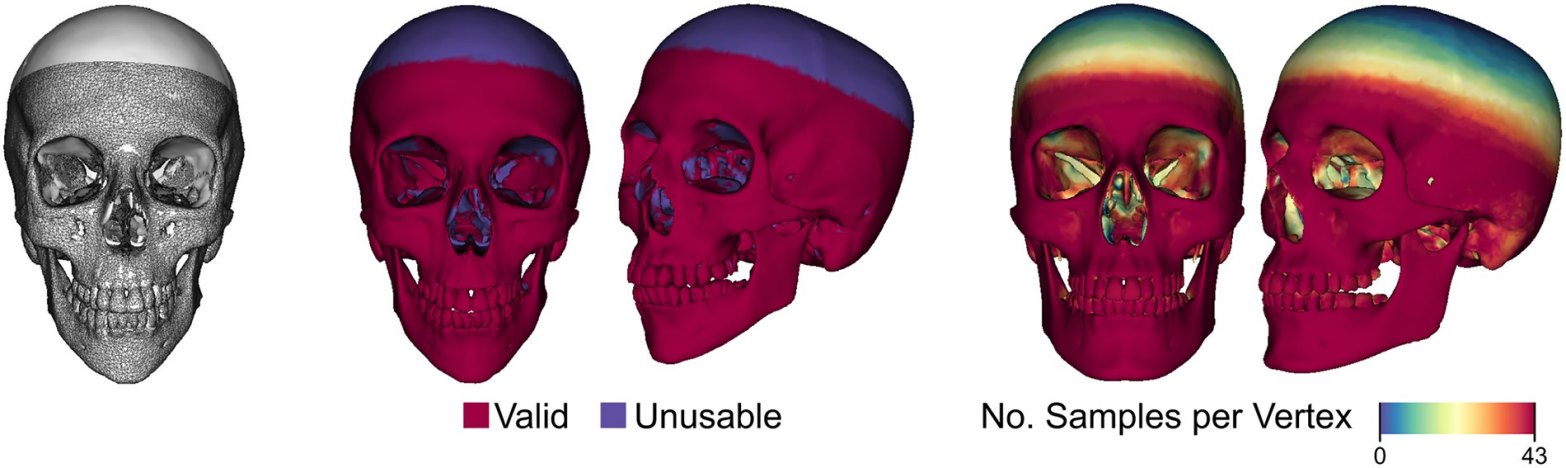
Basis for the statistical evaluation of the FSTT. From left to right: Example of a fitted skull (white) and corresponding extracted skull (black wireframe), validation mask (corresponding to left), number of samples used for all vertices in the statistic of FSTT in Fig 3.

To obtain the FSTT data we often register our complete template skull to *partial* input skulls, which, for instance, have holes in the bony structure or a missing upper part of the calvaria. Fig 4 (left) shows an example of our template skull fitted to a partial skull extracted from CT data. To avoid bias caused by false FSTT measurements, we validate if a vertex of a fitted skull corresponds to a surface point on the corresponding extracted partial skull. We exclude all vertices of the former whose distance to the latter is larger than a given threshold (2 mm in our implementation). This results in the validation mask depicted in Fig 4 (center), which is used for the statistical evaluation. The number of FSTT measurements used for a particular vertex in our statistic is visualized in Fig 4 (right). The facial skull is covered predominantly by all 43 samples, whereas the upper part of the calvaria is covered by a few samples only.

**Fig 5:**
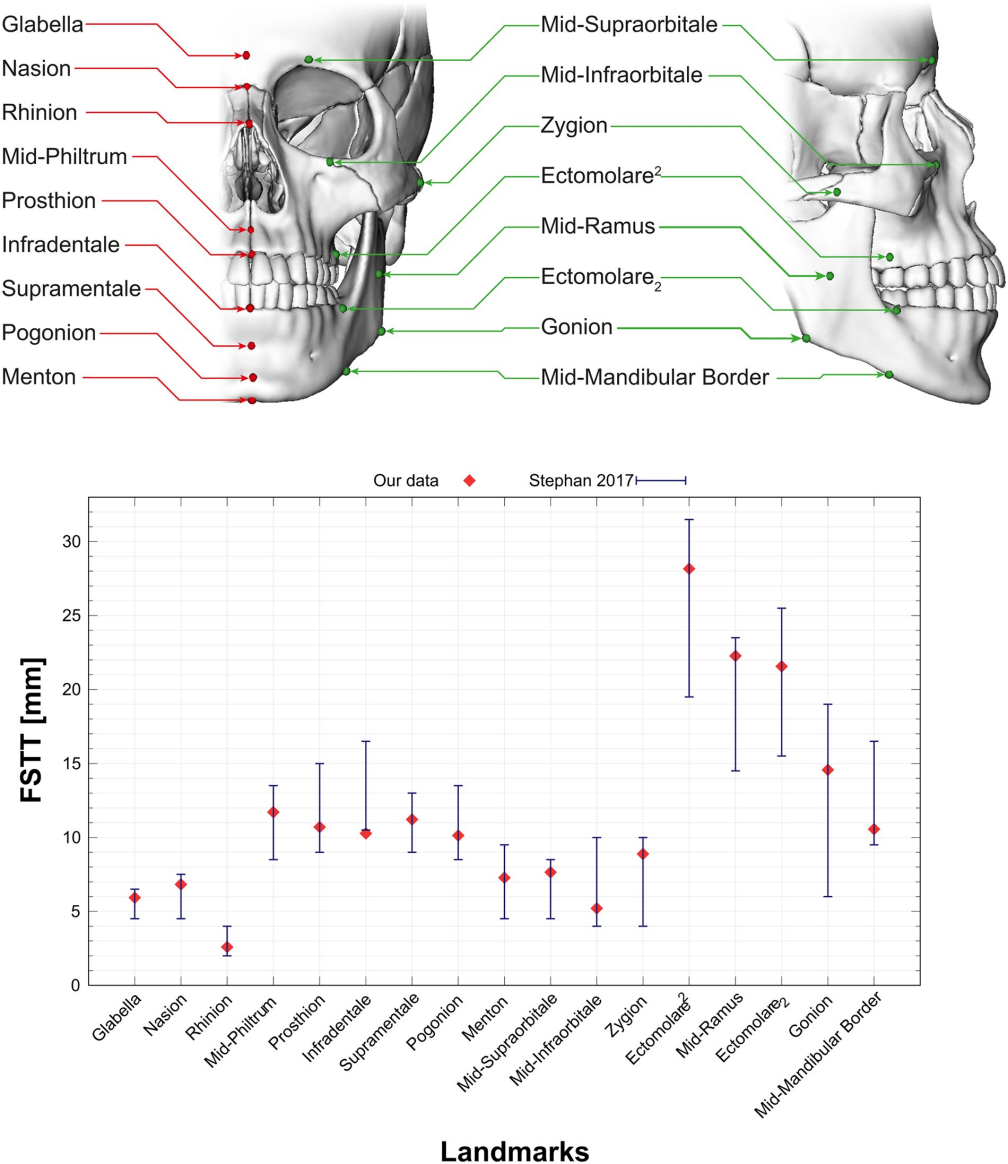
FSTT for commonly used midline and bilateral landmarks. Landmarks defined by [17] as produced by our method (red dots) in relation to pooled data from a recent meta-analysis [18] (weighted mean ± weighted standard deviation as blue error bars).

The generated FSTT statistic is based on 43 different subjects (26 males and 17 females) with a mean age of 28 years. Fig 5 presents the computed FSTT (see Fig 3) at some landmarks commonly used in forensic reconstruction [17]. Our results for these landmarks fit well into the range presented in [18].

### Generating a parametric head model

Similar to the skull model, we generate the parametric head model by fitting a template head to head scans of real subjects, which establishes correspondence between them, and then perform statistical analysis using PCA. For model generation we employ the skin surfaces extracted from the 43 CT scans used for building the FSTT statistics (26 male, 17 female). However, since for some CT scans the nose tip or the upper part of the calvaria are cropped, we bootstrap the model generation by first fitting the template head to a set of 39 optical surface scans (20 male, 19 female) that represent complete heads. We generate a preliminary PCA model from these complete surface scans and use it to fit to the incomplete CT scans, where it fills the missing regions in a realistic manner. The final PCA model is then built from the template fits to all 82 scans.

In the following, a head scan (extracted from CT or generated through optical scan) is represented by its point set $Q=\{q_{1},\ldots ,q_{N}\}$. Since the head models are skin surfaces only, our template head is a surface triangle mesh consisting of *n* ≈ 6 k vertices with positions $H=\{h_{1},\ldots ,h_{n}\}$, as shown in Fig 1. The template fitting process consists of two stages, similar to the skull fitting:

1. We first optimize scaling, rotation, and translation of the template model to align it to the point set Q by minimizing the sum of squared distances between points **q**_*c*_ on the point set Q and their corresponding points **h**_*c*_ on the template model H using ICP [13].
2. After this coarse initialization, we perform a fine-scale non-rigid registration to update the vertex positions H, such that the template model better fits the points Q. Following the approach of [19], we minimize a non-linear objective function
  $$E(H)=E_{\text{fit}}(H)+\lambda_{\text{reg}}E_{\text{reg}}\left(H_{\text{prev}},H\right).$$ (3)

The *fitting term* *E*_*fit*_ penalizes squared distances between points **q**_*c*_ on the point set Q and corresponding points **h**_*c*_ on the template model H:

$$E_{\text{fit}}(H)=\frac{1}{\sum_{c\in C}w_{c}}\sum_{c\in C}w_{c}∥h_{c}-q_{c}{∥}^{2}.$$ (4)

The set of correspondences C consists mostly of *closest point correspondences*, which we construct by finding for each scan point $q_{c}\in Q$ its closest surface point **h**_*c*_ on the template model, and which we filter by pruning unreliable correspondences based on distance and normal deviation thresholds. To allow for more precise fits, we extend these closest point correspondences by 70 *facial landmarks* in the face region, on the ears, and on the lower jaw. These landmarks are manually selected on the template model and on all scans to be fitted (note that this manual work is necessary during model generation only). The per-correspondence weights *w*_*c*_ are used to give the landmarks a higher weight than the closest point correspondences, and to assign a lower weight to surface regions that are not supposed to be fitted closely (e.g., hairs for surface scans or CT artifacts due to teeth restorations).

The *regularization term* *E*_reg_ penalizes the geometric distortion of the undeformed model $H_{\text{prev}}$ (the result of the previous rigid/similarity transformation) to the deformed state H. Since the template head is a surface mesh, we employ a discrete surface deformation model that minimizes bending, discretized by the squared deviation of the per-edge Laplacians

$$E_{\text{reg}}\left(H_{\text{prev}},H\right)=\frac{1}{\sum_{e\in E}A_{e}}\sum_{e\in E}A_{e}∥\Delta^{e}h\left(e\right)-R_{e}\Delta^{e}h_{\text{prev}}\left(e\right){∥}^{2}.$$ (5)

Here, *A*_*e*_ is the area associated to edge *e*, and **R**_*e*_ are per-edge rotations to best-fit deformed and undeformed Laplacians (see [20] for details). In the spirit of non-rigid ICP [19] we alternatingly compute correspondences and minimize (3), starting with a rather stiff surface that is subsequently softened (by reducing λ_reg_) to allow for more and more accurate fits. Whenever λ_reg_ is decreased, we also update the rest state $H_{\text{prev}}$ by the current deformed state H.

From the 39 fits to the complete optical surface scans we construct a preliminary parametric head model. Similar to the skull model generation, we stack the vertex positions of each fitted head **h** = (*x*_1_, *y*_1_, *z*_1_, …, *x*_*n*_, *y*_*n*_, *z*_*n*_)^⊤^ and compute a PCA model of dimension *d* (*d* = 30 in our case), such that we can write

$$H\left(b\right)=\bar{h}+Vb,$$ (6)

where $\bar{h}$ is the mean head, **V** is the matrix containing the principal components in its *d* columns, and **b** = (*β*_1_, …, *β*_*d*_) contains the PCA parameters representing the head.

With the preliminary PCA model at hand, we can now fit the template head to the incomplete skin surfaces extracted from CT scans, where regions of missing data are filled realistically by the PCA model. Fitting to a point set Q amounts to additionally optimizing the PCA parameters **b** during the initial rigid/similarity transformation step. To this end, we minimize squared distances of corresponding points, with a Tikhonov regularization ensuring plausible weights:

$$E_{\text{PCA}}\left(b\right)\;=\;\frac{1}{\sum_{c\in C}w_{c}}\sum_{c\in C}w_{c}∥{\bar{h}}_{c}+V_{c}b-q_{c}{∥}^{2}+\frac{\lambda_{\text{tik}}}{d}\sum_{k=1}^{d}(\frac{\beta_{k}}{\sigma_{k}}{)}^{2}.$$ (7)

In the fitting term, **V**_*c*_ and ${\bar{h}}_{c}$ are the rows of **V** and $\bar{h}$ representing the point **h**_*c*_ corresponding to **q**_*c*_, that is $h_{c}={\bar{h}}_{c}+V_{c}b$. We use λ_tik_ = 1 ⋅ 10^−4^ for the regularization term, where $\sigma_{k}^{2}$ is the variance of the *k*th principal component. The optimal weights **b** are found by solving the linear least-squares problem (7). In step (1) of the head fitting process we optimize for alignment (scaling, rotation, translation) and for shape (PCA weights) in an alternating manner until convergence. Step (2), the non-rigid registration, is then performed the same way as without the PCA model.

**Fig 6:**
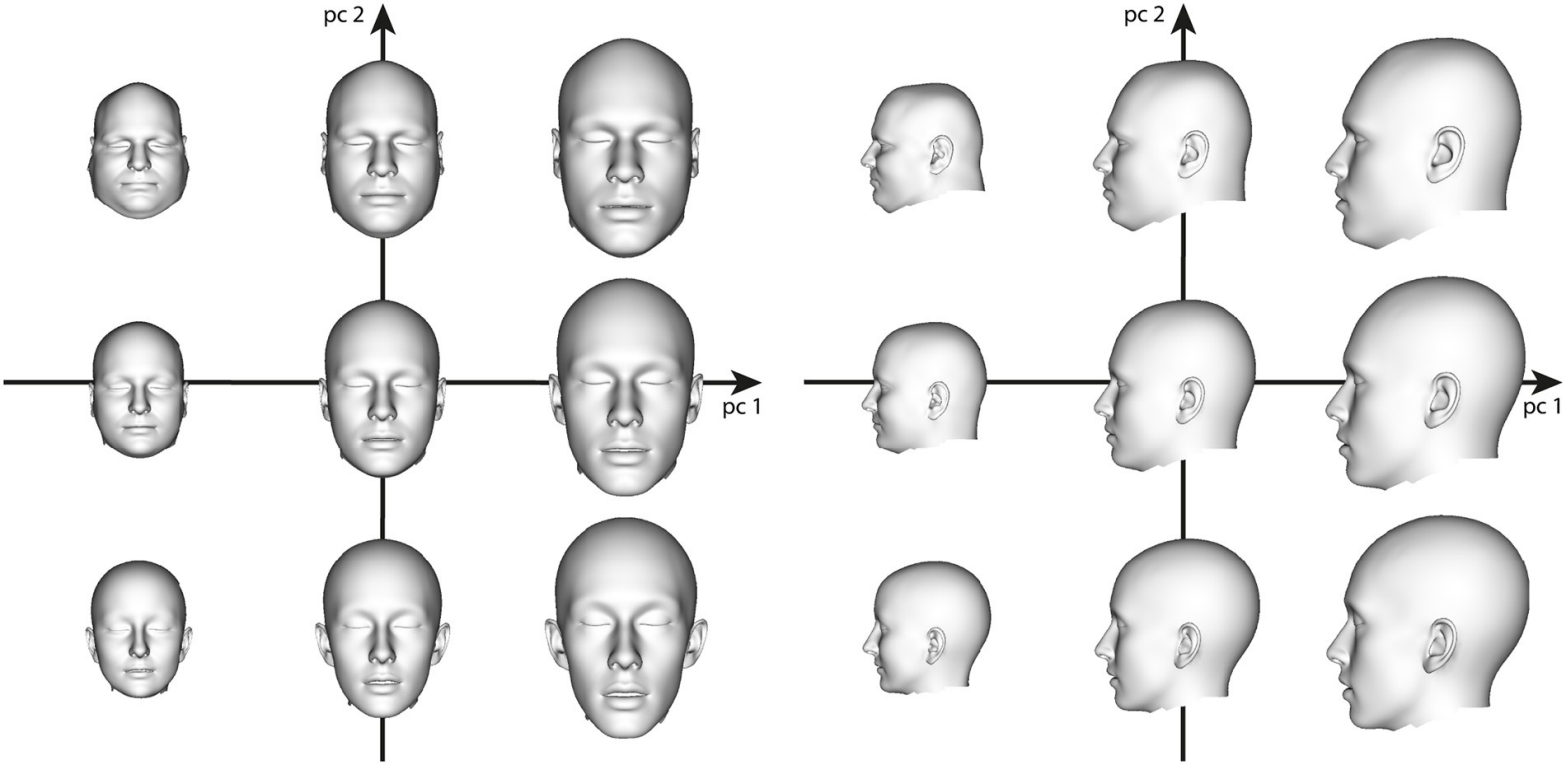
Head variants along the two principal components with the largest eigenvalues. We visualize $\bar{h}+\beta_{1}v_{1}+\beta_{2}v_{2}$, where *β*_*i*_ = *b*_*i*_ ⋅ *σ*_*i*_, *i* = 1, 2, is the weight containing the standard deviation *σ*_*i*_ to the corresponding eigenvector **v**_*i*_, and the factor *b*_*i*_ ∈ {−2, 0, 2}.

We finally combine the fits to the 43 CT scans and to the 39 surface scans into a single parametric PCA head model. The variation of this model along the first two principal directions is shown in Fig 6. While the first principal component basically characterizes head size, the second principal component describes strong variation of head shape within our training data.

In order to analyze the accuracy of our head fitting process, we evaluate the RMS error for all 82 head scans:

$$\text{rms}\left(H,Q\right)=\sqrt{\frac{1}{\sum_{c\in C}w_{c}}\sum_{c\in C}w_{c}∥h_{c}-q_{c}{∥}^{2}}.$$

This is similar to (4) and measures the distance between corresponding point pairs from H and Q. Depending on our input data, we weight down regions that should not be fitted closely (hairs, CT artifacts), such that these regions do not influence the error measure too much. Averaging this error over all 82 scans gives an overall fitting error of 0.19 mm. Note that we prune unreliable correspondences above a distance threshold of 2 mm, which therefore are not considered for error evaluation. However, since the overall fitting error is an order of magnitude smaller, it is not significantly influenced by this pruning.

As done before for the parametric skull model, we also manually select 10 corresponding landmarks on the parametric head model, which are used for the automatic forensic facial reconstruction.

## Automatic forensic facial reconstruction

**Fig 7:**
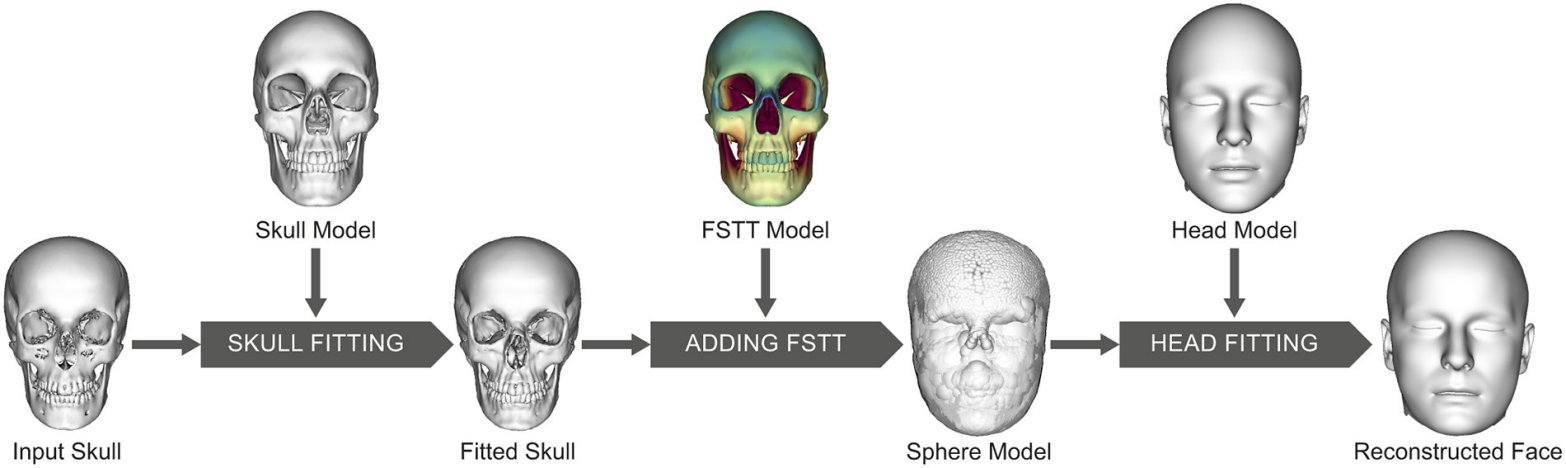
Processing steps of the automatic forensic facial reconstruction. The reconstruction of a face from a given input skull utilizing the generated parametric skull model, the statistic of FSTT, and the parametric head model.

Our automatic forensic facial reconstruction process is based on the generated parametric skull model, the statistic of FSTT, and the parametric head model, described in the previous sections. In the following, we use an anonymized CT scan of a female subject with an age of 21 years to demonstrate the quality of our forensic facial reconstruction. This CT scan was not used for constructing the parametric skull model, head model, or FSTT statistic. The reconstruction process runs in three steps as shown in Fig 7 and is explained in the following sections.

### Skull fitting

Given scanned skull remains as input, the *skull fitting* process is very similar to the registration process described in the section about generating the parametric skull model. The main difference is that we are finally able to utilize the generated parametric skull model (2) as a starting point for the subsequent deformation steps. First, we compute a shape-preserving transformation which aligns the parametric skull model to the given skull by using the global registration approach presented in [12]. To further optimize the alignment we search for reliable point correspondences C between the given skull and the parametric skull model and compute the optimal scaling, rotation, and translation in closed form [21]. After optimizing the alignment, we continue with optimizing the shape. Similar to the PCA fitting of heads (7) we are looking for the coefficient vector **a** of the parametric skull model (2) with

$$E_{\text{PCA}}\left(a\right)\;=\;\frac{1}{\left|C\right|}\sum_{c\in C}∥{\bar{s}}_{c}+U_{c}a-p_{c}{∥}^{2}+\frac{\lambda_{\text{tik}}}{d}\sum_{k=1}^{d}(\frac{\alpha_{k}}{\sigma_{k}}{)}^{2},$$ (8)

where λ_tik_ = 1 ⋅ 10^−3^, $\sigma_{k}^{2}$ is the variance of the *k*th principal component *k* of the skull model and *d* is the number of employed PCA components. Optimization for alignment and shape is alternated until convergence, and before each optimization (alignment or shape) we recompute point correspondences C. After this initialization, we continue with non-rigid registration by minimizing (1).

### Adding facial soft tissue thickness

**Fig 8:**
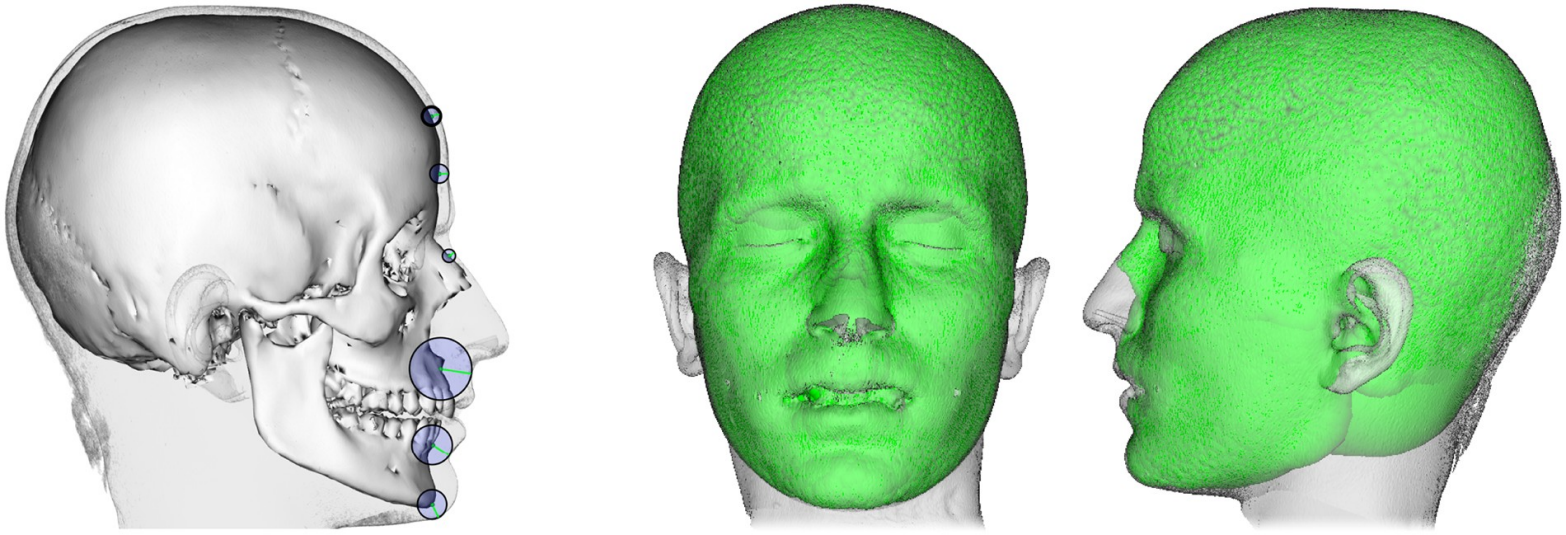
FSTT for a given individual visualized as sphere model. At each skull vertex a sphere with radius of the actual FSTT value from the ground truth data set is drawn. From left to right: Some example spheres for points on the midline, union of all spheres (in green) with original skin surface as overlay.

Next we assign FSTT values based on our FSTT statistic to the fitting result of a given skull. An important advantage of our approach is that our FSTT statistics only contains *scalar* FSTT values without a particular measurement direction, such as skull normal or skin normal, since these directions are hard to determine in a robust manner due to noise or fitting errors. In our case the measured skin position, which is the closest point on the skin surface for a vertex of the skull, is located on a sphere centered at the skull vertex with radius being the corresponding FSTT value. Fig 8 (left) shows a side view of the FSTT measurement results for few preselected points on the midline.

Knowing both the skull and the skin surface for a subject allows the computation of the *actual* FSTT. Fig 8 (center and right) shows an overlay of the extracted skin surface and the union of all spheres centered at the skull vertices and having as radii the appropriate FSTT values, which we call the *sphere model*. The depicted sphere model is based on the exact FSTT of this subject and provides a visually good approximation of the real skin surface. Certainly, since nose and ears do not have a directly underlying bony structure, this method does not provide this kind of information. Approaches for prediction of nasal morphology, such as [22, 23], give some hints about the nose, e.g., the approximated position of the nose tip, but do not really create an individual nose shape for a particular subject. In a real application scenario the age, sex and ancestry of the individual are derived from its skeleton remains and a disaggregated FSTT statistic is used for reconstruction. In our case the sample size is too small to build specific FSTT statistics, so as an approximation we simply build the sphere model based on the mean of our general FSTT statistics (cf. Fig 7).

### Head fitting

**Fig 9:**
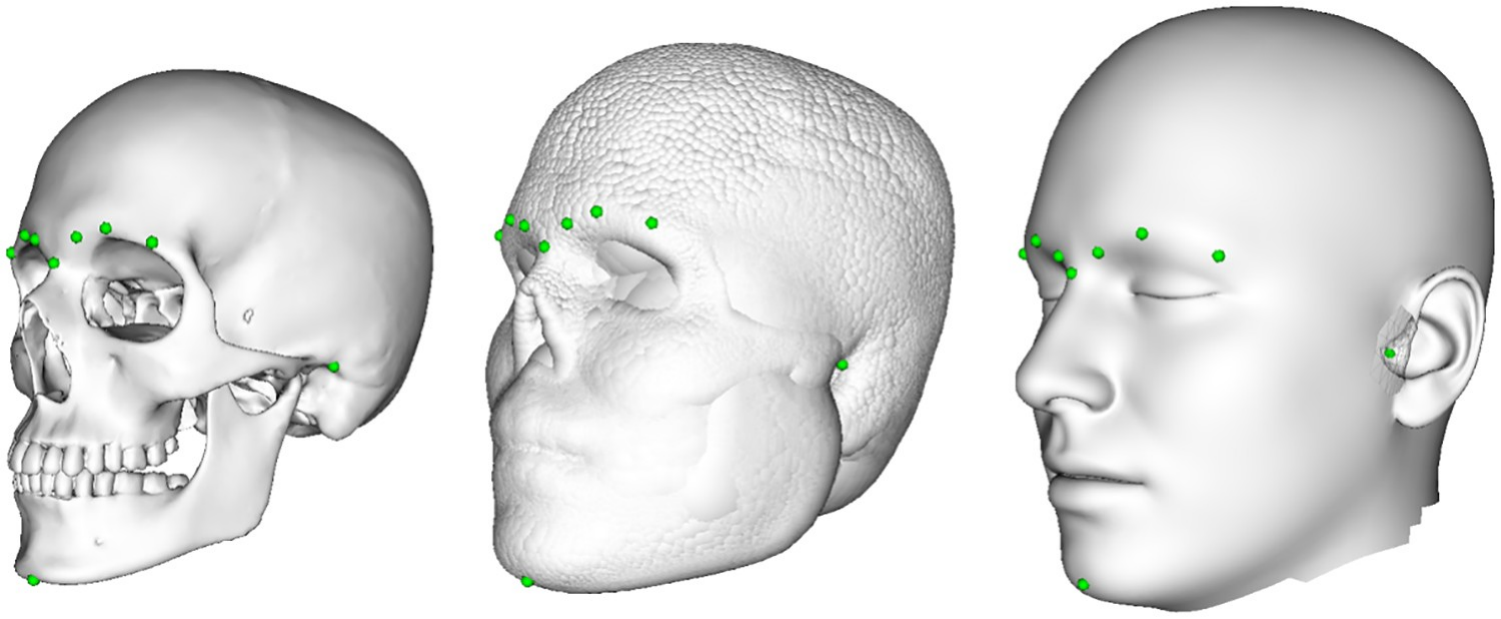
Landmarks for the automatic facial reconstruction. From left to right: Mean skull with preselected landmarks, sphere model based on mean FSTT with projected landmarks, and mean head with preselected landmarks. The landmarks consist of two midline landmarks and four bilateral landmarks, which are selected once on the parametric skull and head model after model generation. The landmarks are based on the proposed nomenclature of [17]: *nasion* and *menton* (from craniometry) and *mid-supraorbitale* and *porion* (from craniometry) as well as *ciliare lateralis* and *ciliare medialis* (from capulometric) and their corresponding counterparts on skull respectively skin surface.

Given a specific sphere model, the next step is to derive a facial profile from this data. For this purpose we deform our parametric head model to the (under-specified) sphere model. The fitting procedure is very similar to the generation of our parametric head model. Similar as before, we initially align the sphere model with the parametric head model. However, this time the landmarks on the fitted skull, which have been selected during the skull model generation, are projected automatically onto the surface of the sphere model as depicted in Fig 9.

The projected landmarks give us robust correspondences on the parametric head model. They are automatically determined and replace the manually selected landmarks used during model generation. We start by optimizing scaling, rotation, and translation, as well as PCA parameters based on the set of landmarks. This initialization is followed by a fine-scale non-rigid registration based on landmarks and closest point correspondences between the parametric head model and the given sphere model.

While this process is very similar to the model generation phase, it differs in the following point: We use the per-correspondence weights *w*_*c*_ in the fitting energy (4) to give points on the outer surface of the sphere model more influence than points in the interior, since the former can be considered as an approximation to the skin surface that we intend to fit. To this end, we first identify if a point **q**_*c*_ on the sphere model is outside from its corresponding point **h**_*c*_ on the template head by checking $n_{c}^{⊤}\left(q_{c}-h_{c}\right)\geq 0$, where **n**_*c*_ is the normal vector of **h**_*c*_. For such correspondences, we set *w*_*c*_ = 1 + 10^8^ ⋅ ∥**h**_*c*_ − **q**_*c*_∥/*B*, where *B* is the bounding box size of model.

As mentioned before, nose and ears do not have a directly underlying bony structure. Thus the sphere models do not provide any data for such regions. Utilizing a parametric head model allows the reconstruction of nose and ears in a statistical sense, i.e., as an element related to the underlying PCA space.

### Generating plausible head variants

**Fig 10:**
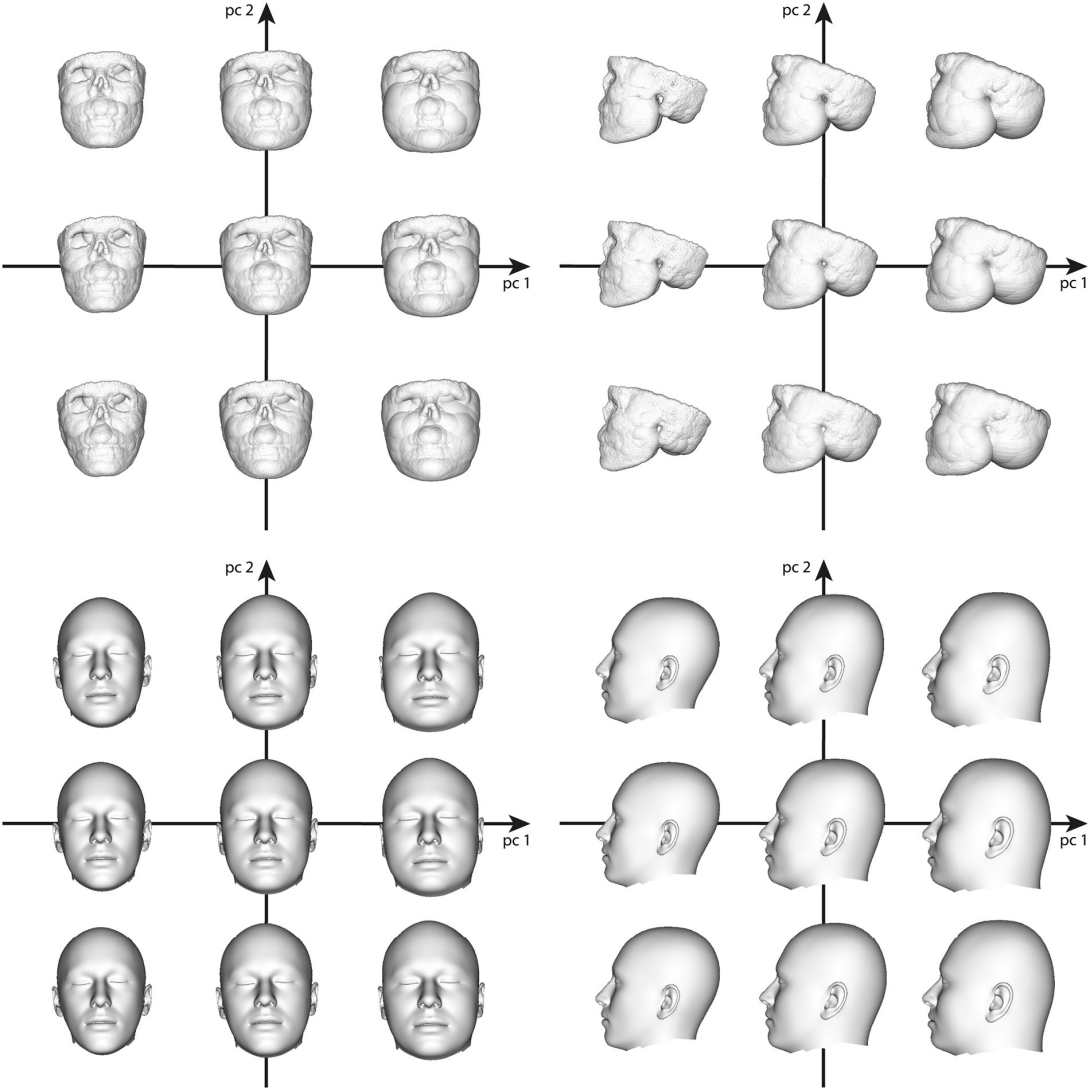
Variants of plausible FSTT distributions for the anonymized given skull. Top: Partial sphere model variants along the two principal components with the largest eigenvalues: We visualize $\bar{t}+\gamma_{1}w_{1}+\gamma_{2}w_{2}$, where *γ*_*i*_ = *c*_*i*_ ⋅ *σ*_*i*_, *i* = 1, 2, is the weight containing the standard deviation *σ*_*i*_ to the corresponding eigenvector **w**_*i*_, and the factor *c*_*i*_ ∈ {−2, 0, 2}. Bottom: Head model fitted to these partial sphere models.

The simplest method for facial reconstruction is to fit the template head to a sphere model based on the *mean* of the FSTT statistics. However, this approximation will rarely match a specific subject. To get a reliable FSTT diversification for an individual, we again adopt the PCA approach creating a parametric FSTT model

$$\text{FSTT}\left(c\right)=\bar{t}+Wc$$ (9)

where $\bar{t}$ is the mean FSTT, **W** contains the principal components of the FSTT, and **c** = (*γ*_1_, …, *γ*_*r*−1_) contains the PCA parameters. Using this parametric FSTT model, we can create plausible FSTT variants for the given input skull. Since the CT scans used for the statistic of FSTT are mostly missing the upper part of the calvaria, the FSTT values obtained in this area are mainly very large and invalid. Thus we omit this area for the construction of our parametric FSTT model (9), which results in partial sphere models. Fig 10 (top) depicts a subset of the partial sphere models along the two principal components with the largest eigenvalues for the given input skull.

Our head fitting process described above can be applied to the partial sphere models without special adjustments. As depicted in Fig 10 (bottom) our approach is able to generate plausible head variants based on the corresponding sphere models in Fig 10 (top). As we are using a parametric model of the complete head, the missing parts like nose, ears and especially the skin surface above the calvaria, are reconstructed in a statistical sense, i.e., as an element related to the underlying PCA space.

## Discussion and conclusion

In this paper we presented an automated method based on a parametric skull model, a parametric head model, and a statistic of FSTT for reconstructing the face for a given skull. The models we are using were derived from head CT scans taken from an existing CT image repository and from 3D surface scans of real subjects. Our approach has three main outcomes: (i) a dense map of FSTT (i.e., a soft tissue layer), (ii) a visual presentation of a statistically probable head based on a statistic of FSTT and a parametric head model, and (iii) a method to generate plausible head or face variants, respectively.

The main advantage of our approach over landmark-based FSTT measurements (see references in [18]) is the density of the FSTT map without the need of error-prone normal information. For any vertex of the parametric skull model a FSTT value can be derived from the statistic of FSTT. It is important to note that the statistical evaluation of the FSTT is fully automatic without any manual interaction. This is different from other FSTT assessments based on CT data, which often still rely on error-prone manual measurements (see, e.g., [24]). The fully automated method introduced here can help to generate a more accurate database in the future, largely overcoming the accuracy issues well-known for manual, landmark-based FSTT assessments [8]. However, as our method is based on CT scans, it is still prone to typical artifacts and gravity effects due to supine patient position. Although our statistic of FSTT so far is generated from only 43 CT scans, the data we derived (Fig 5) clearly indicate good agreement with data just recently published in a meta-analysis [18]. If enough appropriate CT scans are available, rapid processing by means of an automated pipeline can aid the creation of a large statistical database. It seems most likely that methods such as the one introduced here constitute the future for the generation of statistical models from 3D medical imagery. Therefore, enlarging the database will be part of our future work to generate a more precise statistic.

A statistic of FSTT plays a significant role in facial approximation [8] and is also an integral part of modern orthodontic treatment planning [24, 25]. For forensic reconstruction, it forms the basis for further steps in the reconstruction process. The advantage of our approach in comparison to other automated methods [3–7] is that our facial reconstruction process is fully automated. The only manual steps done in our approach are during the model generation processes. As mentioned before, our statistic of FSTT is independent of the measurement direction and thus we utilize sphere models in the reconstruction process. Therefore, error-prone strategies such as averaging over normal vectors to define a measurement direction are completely avoided. Moreover, our parametric FSTT model allows us to create plausible head variants in a statistical sense, which do not require any prior knowledge.

Subsequently, future work will concentrate on merging the two pathways (parametric skull and head model) by integrating all statistical information into one combined model. This model could then be used for various purposes, such as forensic applications, demonstrations for medical procedures, yet also for realistic animations in movies.

**Fig 11:**
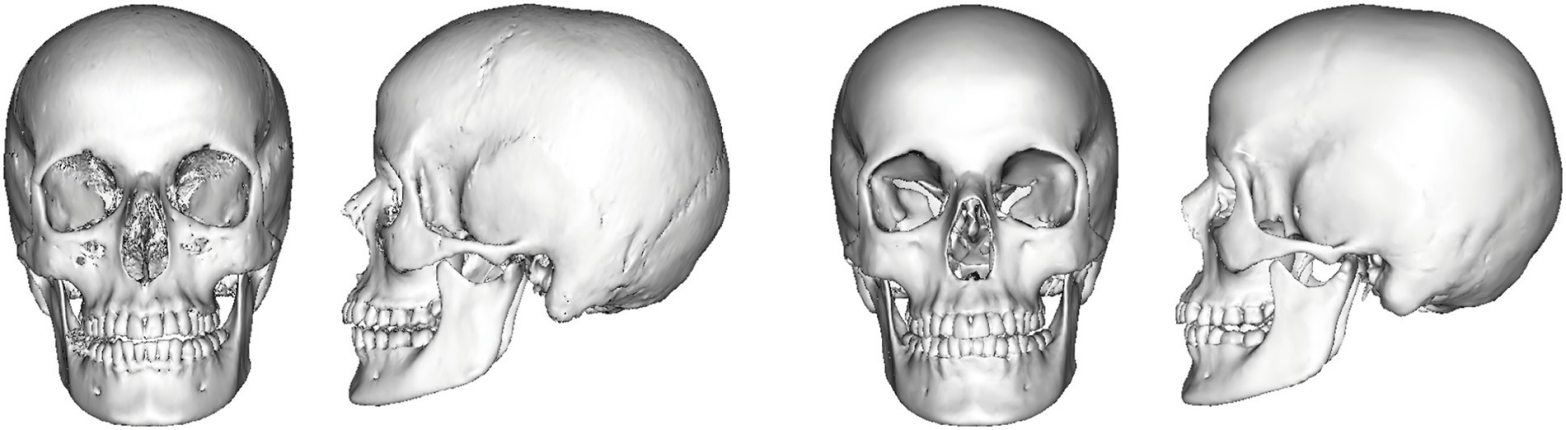
Skull fitting results for a given skull. Extracted skull from CT (left) and fitted skull (right).

**Fig 12:**
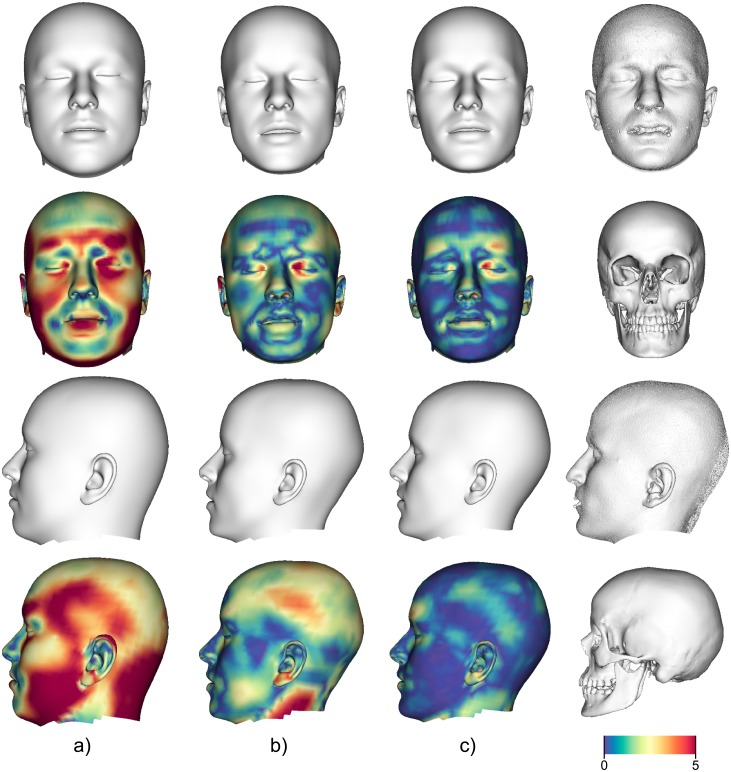
Head fittings with color coded distances (in *mm*) to original skin surface extracted from CT (last column). First three columns from left to right: Fitted head to sphere model based on a) mean FSTT (RMSE 4.04 mm), b) best fit in PCA space (RMSE 1.99 mm), and c) original FSTT (RMSE 1.32 mm).

In conclusion, the automated technique suggested in this paper aids recognition of unknown skull remains (e.g. see Fig 11) by providing statistical estimates derived from a CT head database and 3D surface scans. By creating a range of plausible heads in the sense of statistical estimates, a “visual guess” of likely heads can be used for recognition of the individual represented by the unknown skull. Compared to clay-based sculpturing, which depends on the ability of the operator, our method provides a good approximation of the facial skin surface in a statistical sense (see Fig 12). Nevertheless, the quality of the reconstruction depends on the sample size of the statistic. In order to use additional descriptive factors (e.g., age, sex, ancestry, weight, or skeletal classes [26]), a larger sample size representing the variance of each of the factors is required. We thus aim to enlarge our skull and head database to further elaborate on the method introduced here. Part of our future work is the evaluation of accuracy and recognition of a reconstruction based on our method. Inspired by the approach of Miranda [27], we are planning to collect existing CT datasets and frontal standardized photographs, which are voluntarily donated by subjects for publication and the assessment of accuracy as well as recognition.
